# BB8 Technique: An Innovative Approach for Delivering Diluted Belotero® Balance to Treat Infraorbital Hollows

**DOI:** 10.7759/cureus.111257

**Published:** 2026-06-21

**Authors:** Tuck Wah Siew, Yi Shan Phoon, Lina Pei Shi Yow

**Affiliations:** 1 Dermatology, Radium Medical Aesthetics, Singapore, SGP

**Keywords:** belotero balance, dermal filler, hyaluronic acid, infraorbital hollows, infraorbital rejuvenation

## Abstract

The infraorbital hollow (IOH) and periorbital skin are among the first areas to show signs of facial aging, but are challenging to treat with hyaluronic acid (HA) fillers because of the thin dermis and scant subcutaneous fat. Cohesive polydensified matrix hyaluronic acid (CPM HA, 22.5 mg/mL HA) Belotero Balance gel (CPM-B) is one of the preferred fillers in this region due to its cohesivity and long-held safety reputation. It is typically injected deep into the orbicularis oculi muscle, but adverse events (AEs), including swelling and the Tyndall effect, can still occur. This retrospective, observational three-patient case series describes and evaluates the efficacy and safety of CPM-B delivered superficially (via the novel BB8 technique) in improving the appearance of IOH. Per the BB8 technique, 1 mL of CPM-B was diluted with 0.8 mL of normal saline to achieve an effective HA concentration of 12.5 mg/mL (dilution ratio 1:0.8) and injected superficially to the subdermal plane of the infraorbital region via a cannula. Patients were photographed before and after treatment and followed up regularly for six to nine months post-treatment. IOH severity was graded using the Merz Aesthetic Scale (MAS), while perceived aesthetic improvements by physicians and patients were assessed using five‑point physician (physician global aesthetic improvement scale (PGAIS)) and subject (subject global aesthetic improvement scale (SGAIS)) global aesthetic improvement ratings (1 = very much improved to 5 = worse). The BB8 technique demonstrated efficacy in improving the appearance of IOH. MAS for IOH improved by one to two grades, with effects lasting for six to nine months, while perceived aesthetic improvement ratings were at least “much improved” at all follow-up visits. No persistent or serious AEs were reported. The BB8 technique is a safe and straightforward method for IOH rejuvenation, allowing injectors to effectively address this delicate and challenging area.

## Introduction

The first signs of facial ageing are visible in the infraorbital hollow (IOH) [1,2], where facial volume loss causes wrinkles and folds [3,4] and exaggerates the natural curvilinear depressions beneath the eyes. Patients complain of shadows [2,5] and their face having a tired or sad facial appearance [1,6-8]. The periorbital or infraorbital area comprises the mid-cheek, palpebromalar, and nasojugal grooves, as well as the tear trough [9]. With ageing, thinning of the periorbital skin, attenuation of the orbicularis oculi muscle, and deflation or descent of periorbital fat pads contribute to the deepening of the IOH and increased shadowing. 

Both surgical (e.g., lower eyelid blepharoplasty with fat transposition or injections) and non-surgical alternatives (e.g., dermal fillers; treatments using lasers, light, and radiofrequency; or chemical peels and topical agents) [10,11] are effective and routinely offered treatments for the rejuvenation of the infraorbital region. Hyaluronic acid (HA) dermal fillers have gained significant popularity as a non-surgical, minimally invasive cosmetic treatment to restore volume and support in the periosteal plane for the rejuvenation of the infraorbital region [1,2,7,12,13,14]. The popularity of HA fillers pertains to their ability to deliver natural-looking results, which supports high patient satisfaction [11]. In addition, they have a long-established reputation for safety, relating to their temporary nature and ability to be dissolved with hyaluronidase, if needed [9,10,15-18], thus avoiding the inconvenience and risks associated with surgery [9,10,15]. 

The infraorbital region is challenging to treat due to its thin skin, complex anatomy, minimal soft tissue support, and high aesthetic sensitivity [11,12,13]. Common inadvertent AEs in the region include nodules, swelling, overcorrection, and the Tyndall effect, which typically occur from the superficial placement of HA gels given the thin soft tissue support [5]. As such, the current best practice is to inject fillers deep into the orbicularis oculi muscle in the supraperiosteal plane of the infraorbital region [19]. Consequently, a precise technique and filler selection are essential to minimise the risk of visible irregularities and complications. To address these needs, we aimed to develop a technique for the superficial injection of diluted Belotero Balance® (CPM-B; 22.5 mg/mL HA; Anteis S.A., Plan-les-Ouates, Switzerland, a company of the Merz Aesthetics® group) for IOH correction.

Cohesive polydensified matrix (CPM) HA gel is one of the preferred HA fillers used to treat signs of ageing in the IOH, due to its safety profile with minimal AEs when used in the IOH [20-22]. CPM-B is manufactured using CPM technology, which involves two cross-linking steps leading to the creation of HA gels with high variability in cross-linking densities between constituent HA molecules throughout the gel [23]. CPM HA gels are highly cohesive, homogeneous gels that, upon injection, seamlessly integrate with host tissue. Variability in cross-linking density promotes tissue integration; higher-density zones can fill and expand in tissue spaces, whereas lower-density zones allow the filler to flow and fit into narrower dermal spaces [3,24-27]. CPM-B consists of 22.5 mg/mL of HA suspended within a physiological phosphate buffer containing 0.3% lidocaine hydrochloride. CPM-B recently received approval from the U.S. Food and Drug Administration for treating signs of ageing in IOH [28]. 

While the guidance for treating IOH using CPM-B has typically recommended administering the gel to the supraperiosteal plane, CPM-B is highly cohesive and associated with fewer complications due to superficial product placement [20-22]. The Tyndall effect, presenting clinically as a bluish discoloration when HA filler is placed too superficially, is a complication that injectors seek to avoid in the infraorbital region. In our own practice using CPM gels in appropriate planes, we have found that CPM HA has a low tendency to produce a visible Tyndall effect. These features suggest that CPM-B can be injected more superficially without the same risk of product visibility. The novel “BB8 technique” involves a protocol for treating IOH with superficial placement of diluted CPM-B (1:0.8 ratio with saline, for an effective final HA concentration of 12.5 mg/mL). In our practice, we observed that diluting CPM B with 0.8 mL of normal saline before injection was associated with less post-injection swelling than with the undiluted product placed very superficially. Diluted CPM-B is particularly beneficial when treating the infraorbital area, given the thin nature of the tissue here and the risk of product visibility. This retrospective case series of three patients, all of whom requested improvement of the infraorbital area and facial rhytids, demonstrates the efficacy and safety of the BB8 technique.

## Case presentation

**Figure 1:**
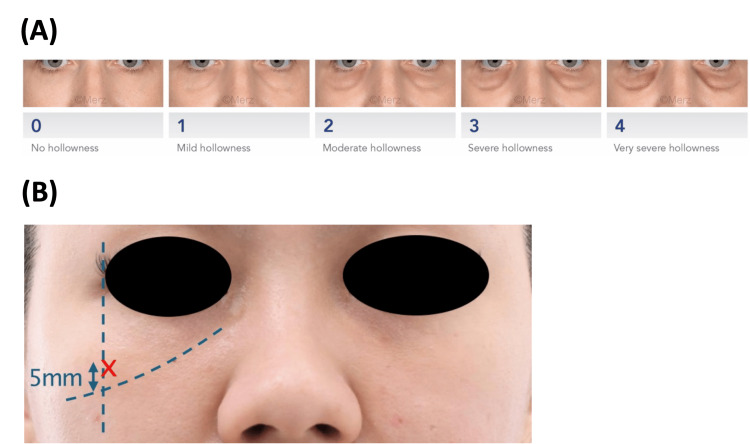
BB8 technique: infraorbital hollow assessment and cannula entry site. (A) Merz Aesthetics Scales for infraorbital hollows - at rest. (B) Cannula entry point marking for the BB8 technique. Reproduced with permission from Merz Aesthetics GmbH. The entry point is marked approximately 5 mm above the intersection of a vertical line from the lateral canthus to the zygomaticocutaneous ligament, with additional markings along the projected course of the infraorbital hollow to guide product distribution.

As part of routine practice, all patients were screened before treatment for prior reactions to anaesthetics, including lidocaine. For this retrospective series, available baseline data included age, sex, qualitative assessment of infraorbital volume loss and pigmentary change, and Merz Aesthetic® Scale (MAS) grading of IOH severity. Patients were photographed at baseline and at follow‑up visits (two weeks, three months, and six to nine months). IOH severity was graded using the MAS, and perceived aesthetic improvement was assessed using physician (physician global aesthetic improvement scale (PGAIS)) and subject (subject global aesthetic improvement scale (SGAIS)) global aesthetic improvement scales. MAS is a validated five‑point photonumeric scale for IOH (MAS Grade 0: no hollowness, to Grade 4: very severe; Figure 1A) [29]. PGAIS and SGAIS are each scored on a five‑point ordinal scale from 1 (very much improved) to 5 (worse) to capture overall aesthetic changes compared with baseline. MAS and PGAIS scores were assigned by the treating physician at each follow‑up visit using standardised photographs and in‑person assessment, while SGAIS scores were reported directly by patients. At each visit, the treating physician also recorded adverse events (AEs) descriptively based on patient reports and physical examinations.

BB8 technique

For each treatment session, one prefilled syringe of Belotero Balance (CPM‑B; 22.5 mg/mL hyaluronic acid) was connected to a 1 mL syringe containing 0.8 mL of preservative‑free normal saline using a sterile connector. The contents of both syringes were passed back and forth three to four times to gently mix the filler and saline, yielding a total of 1.8 mL of diluted product (final HA concentration 12.5 mg/mL, dilution ratio 1:0.8), which was then decanted into three 0.6 mL syringes for injection. All infraorbital injections were performed using a 25G, 40 mm blunt‑tip cannula, which provided a good balance between safety, stiffness, and patient comfort and allowed reliable access to all intended treatment areas. Patients were positioned upright with the head slightly elevated for better appreciation and assessment of the infraorbital hollows. After antiseptic preparation, the cannula entry point (Figure 1B) was marked approximately 5 mm above the intersection of a vertical line from the lateral canthus to the zygomaticocutaneous ligament, with additional markings along the projected course of the infraorbital hollow to guide product distribution. A small pilot opening was created at the entry point with a fine needle, and the cannula was then advanced in the superficial subcutaneous plane, immediately deep to the dermis and superficial to the orbicularis oculi muscle, with the tip and shaft remaining faintly visible through the skin to confirm the superficial position. Diluted CPM‑B was injected using a retrograde linear‑threading and/or fanning technique in tiny droplets, with a very slow injection speed (maximum ~0.1 mL per 15 seconds) and multiple gentle passes while the injector observed the hollow for progressive improvement. In practical terms, this corresponded to ~0.05-0.2 mL per retrograde thread, tailored to baseline hollow severity and skin thickness. The product was distributed both medially and laterally along the infraorbital hollow to achieve a smooth, continuous contour, avoiding bolus injections and overcorrection, and the total volume per side was individualized and recorded. Gentle manipulation optimized the contour and minimized bruising, and the treated area was checked at rest and with animation to confirm the absence of irregularities, overfilling, or visible product. Standard post‑treatment care instructions were provided, including advice on cold compresses, avoidance of pressure on the treated area, and monitoring for AEs. 

Case 1

**Figure 2:**
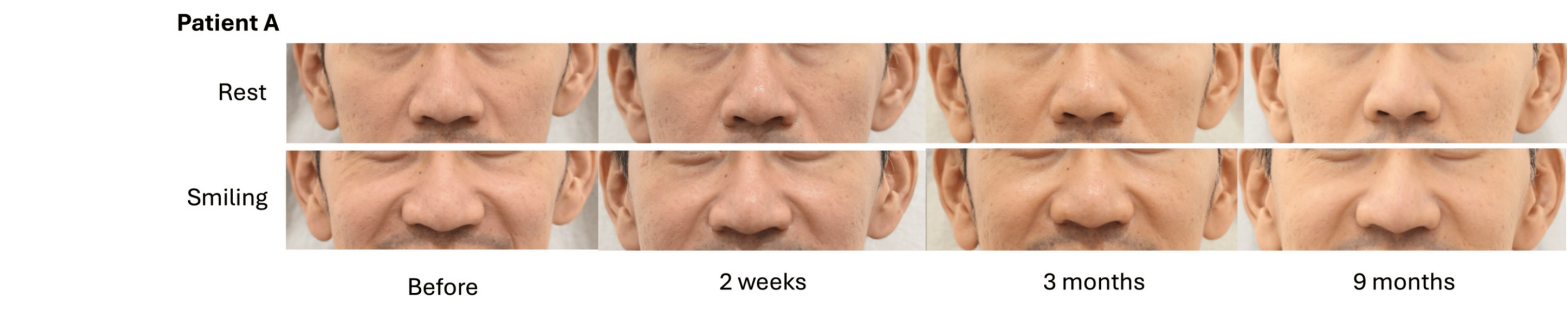
Patient 1 treated with Belotero® Balance using the BB8 technique. Patients photographed at rest (top row) and smiling (bottom row), before (far-left vertical pairs), two weeks (centre-left vertical pairs), three months (centre-right vertical pairs), and nine months (far-right vertical pairs) post-treatment. Visible improvements in IOH were sustained for at least six months. IOH: infraorbital hollow

A 42-year-old male presented with IOH rated as grade 3 (severe) on the MAS. A total of 0.9 mL of diluted CPM-B was injected bilaterally (0.5 mL left; 0.4 mL right). Follow-up assessments were performed at two weeks, three months, and nine months using the MAS for IOH and PGAIS and SGAIS ratings to capture perceived overall aesthetic changes as compared to baseline. By two weeks, the patient’s IOH had improved by two grades to MAS grade 1 (mild), with results maintained through nine months (Figure 2, Table 1). Both the PGAIS and SGAIS were consistently “very much improved” across all follow-up visits, reflecting stable and concordant outcomes.

**Table 1:** Patient 1 treatment ratings after treatment with Belotero® Balance using the BB8 technique. Ratings for the MAS (0: no hollowness/minimal, to 4: very severe) and the physician (PGAIS) and subject (SGAIS) global aesthetic improvement scales (1: very much improved, 2: much improved, 3: improved, 4: no change, 5: worse). pGAIS, physician and subject global aesthetic improvement scales; sGAIS, subject global aesthetic improvement scales; MAS, Merz Aesthetic Scales; N.A., not applicable

Follow-up	Before	Two weeks	Three months	Nine months
MAS	3	1	1	1
PGAIS	N.A.	Very much improved	Very much improved	Very much improved
SGAIS	N.A.	Very much improved	Very much improved	Very much improved

Case 2

**Figure 3:**
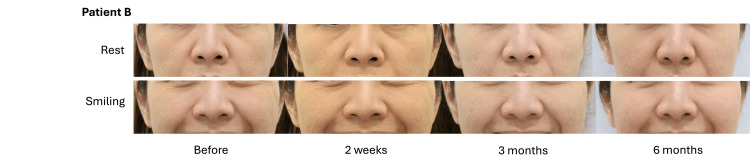
Patient 2 treated with Belotero® Balance using the BB8 technique. Patients photographed at rest (top row) and smiling (bottom row), before (far-left vertical pairs), two weeks (centre-left vertical pairs), three months (centre-right vertical pairs), and six months (far-right vertical pairs) post-treatment. Visible improvements in IOH were sustained for at least six months. IOH: infraorbital hollow

A 48-year-old female (Figure 3) presented with dark eye circles, infraorbital pigment deposition, mild volume loss, and an IOH MAS severity of Grade 2 (moderate) at baseline. A total of 0.6 mL of diluted CPM-B was administered bilaterally (0.3 mL per side) to the infraorbital region using the BB8 technique. Follow-up assessments at two weeks, three months, and six months post‑treatment demonstrated improvements in IOH MAS grade from 2 (moderate) at baseline to 1 (mild) at two weeks, with results maintained through month 6 (Table 2). Both PGAIS and SGAIS consistently rated outcomes as "much improved" compared to baseline at all visits. 

**Table 2:** Patient 2 treatment ratings after treatment with Belotero® Balance using the BB8 technique. Ratings for the MAS (0: no hollowness, to 4: very severe) and the physician (PGAIS) and subject (SGAIS) global aesthetic improvement scales (1: very much improved, 2: much improved, 3: improved, 4: no change, 5: worse). pGAIS, physician and subject global aesthetic improvement scales; sGAIS, subject global aesthetic improvement scales; MAS, Merz Aesthetic Scales; N.A., not applicable

Follow-up	Before	Two weeks	Three months	Six months
MAS	2	1	1	1
PGAIS	N.A.	Much improved	Much improved	Much improved
SGAIS	N.A.	Much improved	Much improved	Much improved

Case 3

**Figure 4:**
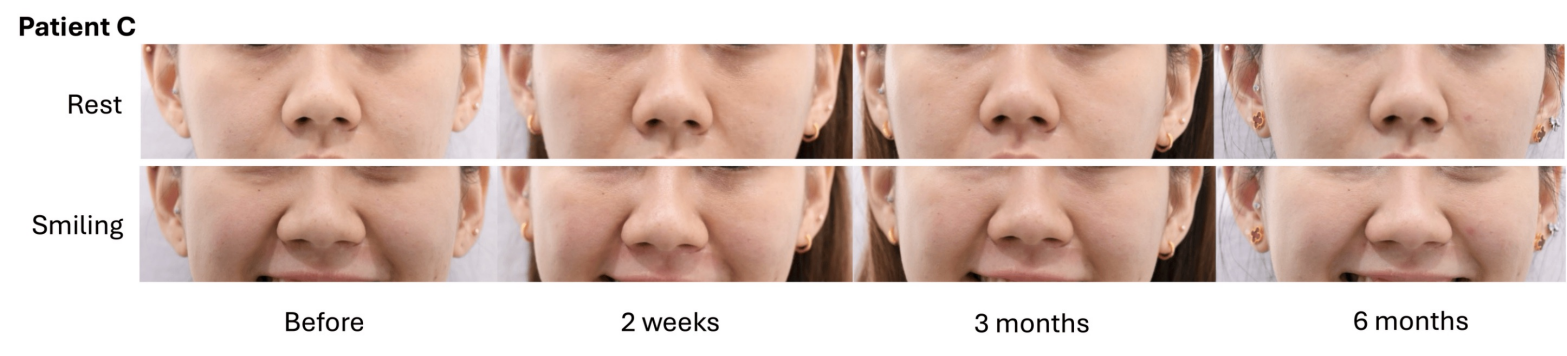
Patient 3 treated with Belotero® Balance using the BB8 technique. Patients photographed at rest (top row) and smiling (bottom row), before (far-left vertical pairs), two weeks (centre-left vertical pairs), three months (centre-right vertical pairs), and six months (far-right vertical pairs) post-treatment. Visible improvements in IOH were sustained for at least six months. IOH: infraorbital hollow

A 28-year-old female (Figure 4) presented with dark eye circles, infraorbital pigment deposition, mild volume loss, and IOH MAS severity of Grade 2 (moderate) at baseline. Treatment was performed using the BB8 Technique, delivering a total of 0.5 mL of diluted CPM-B to bilateral infraorbital regions (left: 0.2 mL, right: 0.3 mL). Similar to Case 2, follow‑up at two weeks, three months, and six months revealed that IOH MAS severity improved from Grade 2 at baseline to Grade 1 (mild) at two weeks, and this improvement was maintained through six months (Table 3). Both PGAIS and SGAIS assessments rated outcomes as "much improved" compared to baseline at all visits, corroborated by clinical photographs.

**Table 3:** Patient 3 treatment ratings after treatment with Belotero® Balance using the BB8 technique. Ratings for the MAS (0: no hollowness, to 4: very severe) and the physician (PGAIS) and subject (SGAIS) global aesthetic improvement scales (1: very much improved, 2: much improved, 3: improved, 4: no change, 5: worse). MAS, Merz Aesthetic Scale; PGAIS, physician global aesthetic improvement scale; SGAIS, subject global aesthetic improvement scale; N.A., not applicable

Follow-up	Before	Two weeks	Three months	Six months
MAS	2	1	1	1
PGAIS	N.A.	Much improved	Much improved	Much improved
SGAIS	N.A.	Much improved	Much improved	Much improved

In all three cases, the results appeared natural without signs of overfilling or facial distortion at rest or in animation. AEs were limited to mild bruising and swelling, which resolved without intervention within two to four weeks. No persistent or serious complications, including Tyndall effect, nodules, or necrosis, were observed.

## Discussion

Our goal was to demonstrate the effectiveness and safety of the novel, simple, and straightforward BB8 technique for treating IOH by the superficial injection of diluted CPM-B. In this three-patient case series, superficial injections of diluted CPM-B yielded sustained aesthetic improvement lasting six to nine months, with strong alignment with subjective ratings across follow-up visits. Importantly, all patients achieved natural-looking outcomes, and only mild, transient adverse events, such as bruising and swelling, were observed. The IOH area is challenging to treat, as the thin and delicate tissue in this region often results in nodules, uneven results, and the Tyndall effect, especially for less experienced injectors. Moreover, the tear trough is an anatomically complex area comprising critical blood vessels and nerves, overlaid by thin skin, and frequently affected by hyperpigmentation, thus complicating the injection of dermal fillers [12]. Treatments, therefore, require a thorough grounding in facial anatomy, as well as expertise with injection techniques. The BB8 technique reduces the HA concentration of CPM‑B, which, in our experience, makes the gel more fluid and easier to handle, facilitating superficial delivery in the infraorbital area. In our practice, this approach was associated with fewer palpable or visible surface irregularities and with natural‑looking outcomes. As the main aim of the BB8 technique is to reduce AEs associated with HA fillers in the infraorbital area, the mild AEs observed by patients in our case series were unsurprising and all resolved spontaneously.

Although the BB8 technique is primarily performed with a blunt‑tip cannula, a needle‑based approach can also be used. In our practice, injection of tiny droplets using a 31G, 8 mm needle attached to a 0.3 mL syringe allows very precise placement but is associated with more bruising than cannula use. For this reason, we generally prefer the cannula technique in the infraorbital region, reserving needle injections for highly selected cases. Overall, we favour the cannula‑based BB8 technique because it offers a favourable balance between precision, safety, and bruising risk compared with the 31G needle approach.

Traditional infraorbital filler techniques typically place product deep on the supraperiosteal plane, which can also yield substantial aesthetic improvement. However, in our clinical practice, these deep injections are more frequently associated with complications such as visible bulging and less natural contours, particularly over time in this highly mobile region where fillers may migrate. By enabling controlled, very superficial placement of a diluted, cohesive gel, the BB8 technique aims to reduce these issues while maintaining effective correction.

This paper introduces the BB8 technique and describes its application for treating IOH using CPM-B in three patients. Outside this formal case series, we have applied the BB8 technique in more than 200 patients (>500 infraorbital treatment areas) in routine practice. In this broader, unpublished experience, we have not encountered persistent or clinically significant irregularities such as sausage‑like protrusions; transient, mild post‑injection swelling similar to that reported in the present three cases is occasionally seen. These observations are anecdotal and were not collected or analysed systematically. The application of diluted CPM-B to the superficial layers, as described in the BB8 technique, has also been useful for the treatment of fine lines and wrinkles in other facial regions, especially in patients and facial locations where the dermal and epidermal layers of the skin are thinner. We now use the BB8 technique to smooth fine lines in the periorbital region (infraorbital, upper eyelid, and lateral canthal) and to treat fine lines in the forehead and neck. The infraorbital skin is very thin and delicate, and undiluted HA fillers, particularly non-CPM HA gels, can absorb excessive water and cause unevenness or the Tyndall effect. The risk of this is reduced with the CPM HA gels due to the high cohesivity and variability in cross-linking density, which are created by the CPM technology [27]. In our experience, a 1:0.8 dilution with normal saline appears to reduce swelling and product visibility when CPM‑B is placed very superficially. It may thus allow for very superficial product placement with a lower likelihood of palpable or visible lumps. We observed that dilution also appears to alter the handling characteristics of the gel, making it subjectively more fluid and easier to distribute evenly for mild volume replacement and skin hydration in the infraorbital area. We also tested dilutions with 0.3 mL, 0.5 mL, and 1.0 mL saline per 1.0 mL CPM‑B, and found that 0.3 mL and 0.5 mL produced clinically relevant swelling in some patients when injected very superficially, whereas 1.0 mL often gave suboptimal correction that required repeat injections and rarely lasted beyond about 3 months. By contrast, 0.8 mL provided a favourable balance between efficacy and tolerability, with minimal post-injection swelling in the infraorbital region, as compared to lower dilutions with 0.3 mL and 0.5 mL of normal saline (data not shown). In the author’s practice, Restylane® Vital (Q-Med AB, Uppsala, Sweden) was previously used for the infraorbital region, and although effective, the outcomes were less natural, and some patients developed the Tyndall effect. Clinically, we also found that patients presenting with significant volume deficiencies in the medial and lateral suborbicularis oculi fat can benefit from additional injections with a CPM HA gel with higher viscosity and elasticity, e.g., CPM-I or CPM-V (Belotero® Intense / Belotero® Volume; Anteis S.A., Plan-les-Ouates, Switzerland, a company of the Merz Aesthetics® group) to provide deeper augmentation and facilitate complete rejuvenation of the midface and infraorbital areas.

This case series was limited by its retrospective nature, uncontrolled design, and small sample size (three patients). The three cases presented were selected from our retrospective records to illustrate the BB8 technique across a range of infraorbital hollow severities in patients with complete six-to-nine‑month photographic follow‑up. The three cases were selected as descriptive examples rather than a consecutive cohort, and no statistical analyses were performed. Our findings should be interpreted cautiously and may not be generalizable to broader or more diverse populations due to potential selection bias. We also did not conduct objective volumetric imaging or skin or pigment measurements. In our experience, commonly used 3D imaging systems (such as QuantifiCare) do not reliably capture subtle changes in the infraorbital region, and we thus relied on standardized clinical photography under consistent white light and background conditions. Future prospective studies incorporating imaging modalities optimized for the infraorbital area could provide more quantitative assessments of volume and skin quality changes. While the outcomes are in line with our broader clinical experience, additional data from a more diverse patient population, including a wider range of IOH severities, may help strengthen and corroborate these findings. Finally, our experience on ease of handling and perceived reductions in unevenness reflects clinical impressions and would benefit from future formal comparative analyses.

## Conclusions

The infraorbital region is a common area in which patients request treatment for aesthetic rejuvenation, but challenging for injectors to treat because of its complex anatomy, thin and delicate tissues, and susceptibility to filler-related irregularities. This case series introduces the novel BB8 technique involving the superficial injection of diluted CPM-B as a safe, effective, and practical method for IOH correction. In our experience, the diluted formulation was associated with smoother injection, homogeneous tissue integration, and less superficial product visibility or swelling when used very superficially in the infraorbital area. The three patients treated in this case series showed visible and sustained improvements in IOH severity lasting up to nine months, with no serious AEs being reported. Taken together, these outcomes provide preliminary data supporting the use of the BB8 technique for treating the infraorbital area, particularly in patients with thin dermis or high risk for complications with traditional filler placement. By diluting CPM-B with normal saline, we present a novel, safe, and straightforward method to rejuvenate IOH, allowing injectors to effectively address this delicate and challenging area.
